# 1110. *In Vivo* Pharmacodynamics of Vancomycin Against Staphylococci in Young Infants

**DOI:** 10.1093/ofid/ofab466.1304

**Published:** 2021-12-04

**Authors:** Amanda Gwee, Stephen Duffull, Derek Zhu

**Affiliations:** 1 The Royal Children’s Hospital, Melbourne, Victoria, Parkville, Victoria, Australia; 2 University of Otago, Dunedin, Otago, New Zealand

## Abstract

**Background:**

Coagulase-negative staphylococci are the predominant pathogen causing late onset sepsis in young infants, however, the pharmacodynamic target for vancomycin therapy is unknown. This study aimed to determine the pharmacodynamic target of vancomycin in young infants with staphylococcal infections.

**Methods:**

Retrospective data were collected for infants aged 0-90 days with methicillin-resistant *Staphylococcus aureus* (MRSA or coagulase-negative staphylococci (CoNS) bacteraemia over a 4-year period at the Royal Children’s Hospital Melbourne, Australia. Vancomycin broth microdilution minimum inhibitory concentrations (MIC) were determined. A published pharmacokinetic model was externally validated using the study dataset and a time-to-event pharmacodynamic model developed using non-linear mixed effects modelling, with the event being the first negative blood culture. Simulations were performed to determine the 24-hour trough vancomycin concentration correlating with a 90% probability target attainment (PTA) of the area under the curve in the first 24-hours (AUC_0-24_) exceeding the identified target.

**Results:**

Thirty infants, 28 with CoNS and two with MRSA bacteraemia, who had 165 vancomycin concentrations determined were included. The vancomycin broth microdilution MIC was determined for 24 CoNS and one MRSA isolate, both with a median MIC of 1 mg/L (CoNS range 0.5 to 4). An AUC_0-24_ ≥3 00 mg/L·h was associated with a 7.8-fold increase in the chance of bacteriological cure for all staphylococci at any time point compared to an AUC_0-24_ < 300 mg/L·h (hazard ratio 95% CI: 3.21-18.8). The 24-hour trough concentrations associated with a 90% PTA of achieving this target were > 13-16 mg/L and > 8-12 mg/L for 6 and 12-hourly dosing, respectively.

**Conclusion:**

Our study found that an AUC_0-24_ ≥ 300 mg/L·h was associated with a 7.8-fold increase in bacteriological cure in young infants with staphylococcal bloodstream infections.

**Disclosures:**

**All Authors**: No reported disclosures

